# Remote cadaveric minimally invasive surgical training

**DOI:** 10.3389/fped.2023.1255882

**Published:** 2023-10-09

**Authors:** Go Miyano, Makoto Takahashi, Takamasa Suzuki, Hisae Iida, Eri Abe, Haruki Kato, Shiho Yoshida, Geoffrey J. Lane, Koichiro Ichimura, Kazuhiro Sakamoto, Atsuyuki Yamataka, Tadaharu Okazaki

**Affiliations:** ^1^ Pediatric Surgery, Juntendo University Urayasu Hospital, Chiba, Japan; ^2^ Coloproctological Surgery, Juntendo University School of Medicine, Tokyo, Japan; ^3^ Pediatric General & Urogenital Surgery, Juntendo University School of Medicine, Tokyo, Japan; ^4^ Anatomy and Life Structure, Juntendo University School of Medicine, Tokyo, Japan

**Keywords:** remote education, minimally invasive surgery, cadaver surgical training, pediatric surgery, general surgery

## Abstract

**Objective:**

The aim of the study is to discuss the efficacy of live vs. remote cadaver surgical training (CST) for minimally invasive surgery (MIS).

**Methods:**

A cohort of 30 interns in their first and second years of training were divided into three groups: live observers (*n* = 12), live participants (*n* = 6), and remote observers: (*n* = 12). The interns had the opportunity to either observe or actively participate in two different surgical procedures, namely, laparoscopic lower anterior resection, performed by a colorectal surgical team, and laparoscopic fundoplication, performed by a pediatric surgical team. The procedures were conducted either at a base center or at a remote center affiliated with the institute. Some of the interns interacted directly with the surgical teams at the base center, and others interacted indirectly with the surgical teams from the remote center. All interns were administered questionnaires before and after completion of the CST in order to assess their understanding of various aspects related to the operating room layout/instruments (called “design”), accessing the surgical field (called “field”), understanding of anatomic relations (called “anatomy”), their skill of dissection (called “dissection”), ability to resolve procedural/technical problems (called “troubleshooting”), and their skill in planning surgery (called “planning”) according to their confidence to operate using the following scale: 1 = not confident to operate independently; 4 = confident to operate with a more senior trainee; 7 = confident to operate with a peer; and 10 = confident to operate with a less experienced trainee. A *p *< 0.05 was considered statistically significant.

**Results:**

All scores improved after CST at both the base and remote centers. The following significant increases were observed: for remote observers: “field” (2.67→4.92; *p *< .01), “anatomy” (3.58→5.75; *p *< .01), “dissection” (3.08→4.33; *p *= .01), and “planning” (3.08→4.33; *p *< .01); for live observers: “design” (3.75→6.17; *p *< .01), “field” (2.83→5.17; *p *< .01), “anatomy” (3.67→5.58; *p *< .01), “dissection” (3.17→4.58; *p *< .01), “troubleshooting” (2.33→3.67; *p *< .01), and “planning” (2.92→4.25; *p *< .01); and for live participants: “design” (3.83→6.33; *p *= .02), “field” (2.83→6.83; *p *< .01), “anatomy” (3.67→5.67; *p *< .01), “dissection” (2.83→6.17; *p *< .01), “troubleshooting” (2.17→4.17; *p *< .01), and “planning” (2.83→4.67; *p *< .01). Understanding of “design” improved significantly after CST in live observers compared with remote observers (*p *< .01). Understanding of “field and “dissection” improved significantly after CST in live participants compared with live observers (*p *= .01, *p *= .03, respectively). Out of the 12 remote observers, 10 participants (83.3%) reported that interacting with surgical teams was easy because they were not on-site.

**Conclusions:**

Although all the responses were subjective and the respondents were aware that observation was inferior to hands-on experience, the results from both centers were equivalent, suggesting that remote learning could potentially be viable when resources are limited.

## Introduction

The postgraduate training program in Japan was revised in 2004. This revision affected graduates of accredited medical schools who had completed 6-year-long courses and successfully passed the national medical registration examination. The previous system, which involved immediate commencement of specialty training after graduation, was replaced with an internship system where all graduates are required to spend the first 2 years after graduation rotating through multiple departments. While the aim of this revision was to train doctors with more general experience and skills, the duration of surgical training was limited to a period of 6 months, which was very different from the former system in which prospective surgeons would immediately commence on their surgical training after graduation.

As a result, surgical trainees under the new system must commence surgical training with only limited exposure to surgery. In recent years, surgical training with training boxes and laboratory animals has increased, resulting in a distinct trend away from direct, hands-on management with some interns having little access to experiencing surgery. One of the primary challenges with utilizing training models and laboratory animals is their physical difference to actual patients; the variety of patients cannot be reproduced adequately with animals, and of course, there are differences in anatomic relations (1–3). Thus, options for improving exposure to surgery are decreasing while the COVID-19 pandemic and social distancing rules have also limited access to in-person training activities and prevented attendance at “live” teaching sessions. As a result, medical education has been forced to evolve rapidly to a virtual format (4) facilitated by improved data transmission. While surgical procedures are often video recorded for educational purposes, such as utilization at congresses/conferences and live digital learning events (5), and some hospitals even broadcasted operations to waiting rooms so relatives can observe the surgery, such options have not been generally applied for routine teaching purposes due to patient safety and ethical concerns associated with live surgery broadcasts (6).

Cadaver surgical training (CST) is an integral part of understanding and learning anatomy and a time-tested technique for obtaining valuable understanding of the structure and textures of the body required for confident surgical intervention. CST would be most beneficial as a “live” experience, but virtual or remote CST may provide exposure that would otherwise be unattainable and could prove to be valuable after COVID-19 related restrictions, such as social distancing and ease (7). Remote CST has been reported for plastic surgery (7), and its potential for minimally invasive surgery (MIS) training is being considered.

In order to provide surgical trainees with exposure to improve their education and understanding of anatomy and aspects of planning and logistics essential for successful surgery, a previous experience with CST and laparoscopy/thoracoscopy (8) was applied to determine the impressions of first and second year interns faced with increasingly limited opportunities for surgical experience exacerbated by COVID-19 restrictions. In this study, groups of interns either observed or participated in CST procedures directly at a base center (live observers/live participants) or observed the same procedures from a remote center at an affiliated institute (remote observers). In addition, their perceived confidence to operate was assessed by questionnaires administered before and after CST. Their responses were considered likely to be useful for assessing the potential of remote education as a viable alternative to direct teaching methods as a means for improving the efficiency of medical education.

## Methods

The Juntendo University CST Center was established in 2019, rendering it accessible to the 18 specialty surgical departments at Juntendo University Medical School Hospital, as well as the Department of Anatomy at Juntendo University Medical School. The center is supported financially by the Japanese Ministry of Health, Labour, and Welfare, for the effective use of donated bodies for the advancement of science (8).

**Figure 1:**
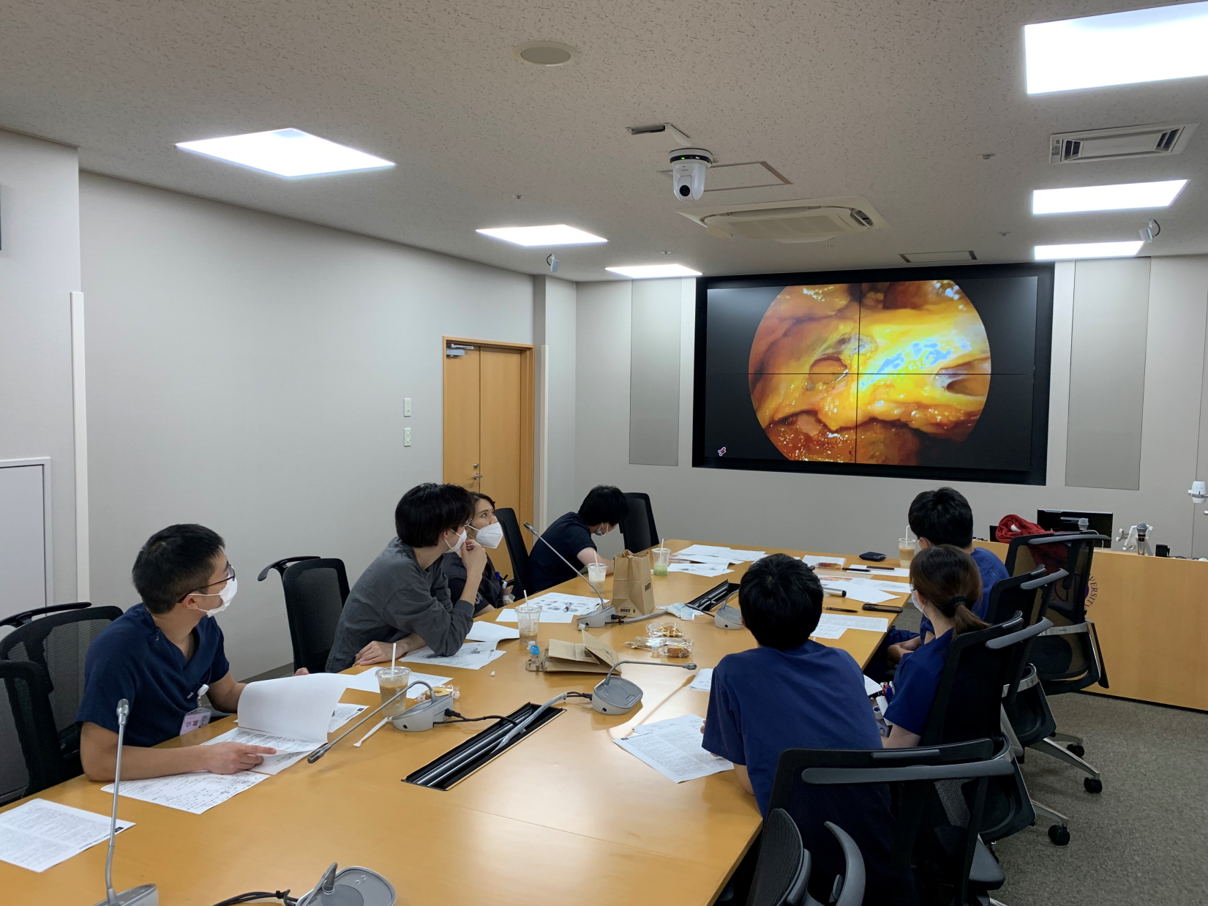
Remote CST observation. First and second year interns participated as observers; they were allowed to ask questions freely during their CST session.

The CST Center located at Juntendo University Hospital (base center) was connected to a remote center at Juntendo University Urayasu Hospital (Figure 1) by a data transmission system established with a “KAKENHI” grant (grant number: 22K02835) from the Japan Society for the Promotion of Science and Kawano Masanori Memorial Public Interest Incorporated Foundation for Promotion of Pediatrics. Juntendo University utilized its own transmission network to offer on-site video and audio broadcast services using highly secure connections to prevent information leakage.

When the CST program was first established at Juntendo, the cadavers were embalmed using a saturated salt solution. For the current study, only cadavers preserved using Thiel's method (9) were used. A total of 30 first and second year interns either observed or participated in a laparoscopic lower anterior resection performed by a colorectal surgical team and a laparoscopic fundoplication performed by a pediatric surgical team directly at the base center (live observers = 12, live participants = 6) or observed the same procedures from the remote center (remote observers = 12). Live participants had the opportunity to alternate roles as the main surgeon, scope surgeon, and assistant surgeon. The two operations used for CST were chosen based on their frequent utilization in clinical practice, involving a variety of maneuvers. All interns were allowed to interact freely with the surgical teams directly at the base center and indirectly at the remote center. All interns involved at both centers were administered a questionnaire before and after their CST session that asked regarding their impressions of six criteria: operating room layout/logistics, including an introduction of trocars, trocar selection, and trocar insertion (called “design”), accessing/establishing the surgical field (called “field”), understanding of anatomic relations (called “anatomy”), understanding of basic dissection techniques (called “dissection”), dealing with procedural/technical problems (called “troubleshooting”), and how to plan surgery (called “planning”). Questionnaires assessed how confident interns with no hands-on experience of surgery would feel about operating, based on the understanding derived from their CST session. The responses were scored as follows: 1 = not confident to operate independently; 4 = confident to operate with a more senior trainee; 7 = confident to operate with a peer; 10 = confident to operate with a less experienced trainee. A sample questionnaire is presented in Table 1.

**Table 1:** Perceived self-confidence.

	Pre-CST	Post-CST
1. Operating theater layout (including trocar selection)		
2. Accessing the surgical field		
3. Understanding anatomy		
4. How to dissect		
5. Solving problems		
6. Planning surgery		

1. Not confident at all; 4. Confident if with someone senior; 7. Confident with peer; 10. Confident to supervise someone junior.

Data were analyzed using standard statistical methods with the software Statcel-2 (OMS Publishing Inc., Saitama, Japan). Technical background of interns was compared using Bonferroni corrected *post hoc* tests. The changes of the score from the questionnaire were compared using the Student's *t*-test. For all statistical tests, *p *< .05 was used to determine significance. Juntendo University institutional review board approval was obtained for this study (2019173). Methodology and ethics were in accordance with the Helsinki Declaration (2013).

## Results

**Figure 2:**
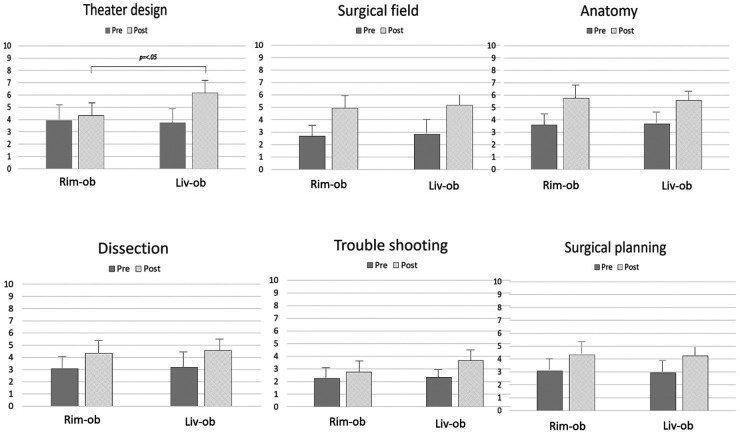
The changes in understanding after CST, comparing the remote and base center interns. How to design an operating theater, how to develop a surgical field, how well anatomy was understood, how to dissect during an operation, how to deal with problems, and how to plan surgery. How to design an operating theater was significantly different between the base and remote center interns.

The technical backgrounds of the first and second year interns are shown in Table 2. There were no significant differences observed among the live observers, live participants, and remote observers. While the scores at both centers increased for all criteria after CST, a significant improvement was reported by the remote observers for the following: “field” (2.67 ± 0.9→4.92 ± 0.9; *p *< .01), “anatomy” (3.58 ± 0.7→5.75 ± 0.9; *p *< .01), “dissection” (3.08 ± 0.9→4.33 ± 0.9; *p *= .01), and “planning” (3.08 ± 1.0→4.33 ± 1.1; *p *< .01); by live observers for all criteria: “design” (3.75 ± 1.2→6.17 ± 1.1; *p *< .01), “field” (2.83 ± 1.1→5.17 ± 0.9; *p *< .01), “anatomy” (3.67 ± 0.7→5.58 ± 0.7; *p *< .01), “dissection” (3.17 ± 1.1→4.58 ± 0.9; *p *< .01), “troubleshooting” (2.33 ± 0.6→3.67 ± 0.9; *p *< .01), and “planning” (2.92 ± 1.0→4.25 ± 0.7; *p *< .01). Only “design” was significantly higher when the rates of increase between live observers and remote observers were compared (*p *< .01). Figure 2 shows the results for the live observers and remote observers. Interestingly, 10 participants out of the 12 (83.3%) remote observers returned the questionnaires with equivalent results to the live observers; seven out of the 12 remote observers (58.3%) were interested in attending another remote CST session.

**Table 2:** Overall dexterity of interns.

		Live observers	Live participants	Remote observers	*p*-Value
		(*n* = 12)	(*n* = 6)	(*n* = 12)	
Surgical field of interest	Gastrointestinal	3	2	4	ns
	Cardiac	1	1	1	
	Hepatobiliary	2	0	1	
	Pediatric	3	2	3	
	Others	3	1	3	
Type of workplace	University hospital	7	3	8	ns
	General hospital	2	1	1	
	Private clinic	1	0	0	
	Others	2	2	3	
Exercise experience	Team sports	8	5	9	ns
	Personal exercise	2	1	2	
	None	2	0	1	
Musical instrumental experience	Member of a group	1	0	1	ns
	Personal pleasure	3	2	4	
	None	8	4	7	
Video game experience	Regular player	2	2	3	ns
	Occasional player	8	3	7	
	None	2	1	2	

**Figure 3:**
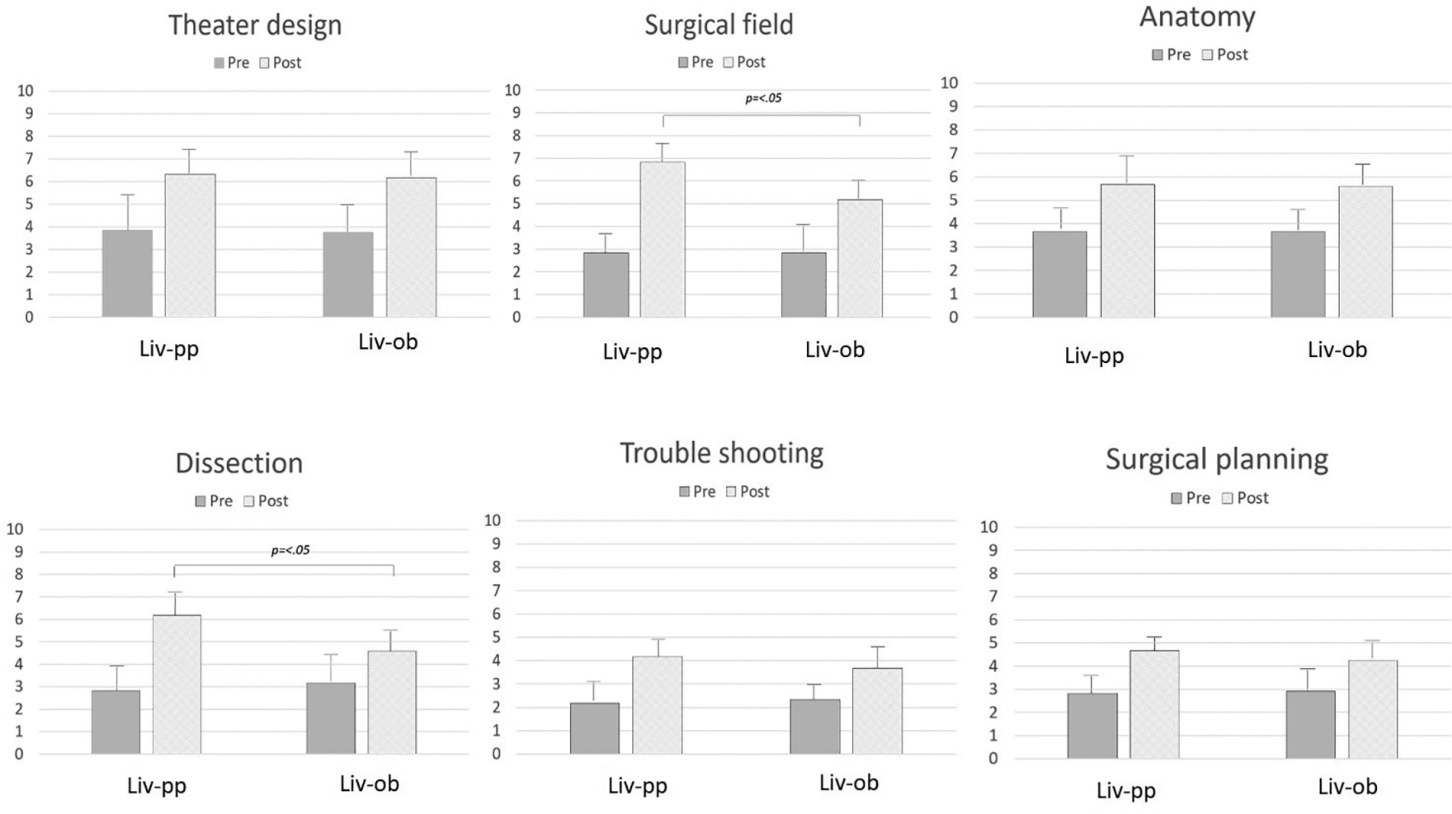
The changes in understanding after CST, comparing the live observers and live participants. How to access a surgical field and how to dissect during an operation were significantly different between the live observers and live participants.

For the live participants, significant increases were observed in the scores after CST for “design” (3.83 ± 1.7→6.33 ± 1.0; *p *= .02), “field” (2.83 ± 0.7→6.83 ± 0.7; *p *< .01), “anatomy” (3.67 ± 0.8→5.67 ± 0.8; *p *< .01), “dissection” (2.83 ± 1.1→6.17 ± 1.3; *p *< .01), “troubleshooting” (2.17 ± 0.7→4.17 ± 0.7; *p *< .01), and “planning” (2.83 ± 0.7→4.67 ± 0.5; *p *< .01). “Field” and “dissection” increased significantly in the live participants, reflecting the benefit of hands-on experience (*p *= .01, *p *= .03, respectively). Figure 3 shows the results for the live observers and live participants.

## Discussion

The recent report (8) on CST for MIS training examined its relevance and the extent to which CST was considered realistic. The findings of this study were applied when designing the current study to establish the goals for training and how to assess the sense of achievement after CST at both the base and remote centers. The impressions of CST at both centers were comparable, with the remote observers particularly supportive of their remote learning experience. McIntyre et al. (10) compared the subjects observing surgery in an operating theater with subjects watching a live broadcast of the same surgery; the group engaged in live broadcast asked four times more questions than the group in the operating theater, and more of their questions were answered. There was a similar trend in this study with the remote observers interacting more during CST than the live observers/live participants, possibly related to not being physically close to the surgeons teaching the CST session on-site. As all interns considered CST as a valuable learning experience, easier interaction would be advantageous and a possible reason why the remote observers found their experience so worthwhile and actively chose “remote” as the format for a repeat CST session. In other words, a virtual cadaver experience provided exposure and an opportunity to learn that was less stressful than being with the instructors directly. The remote observers felt more relaxed due to their physical separation from the senior staff, which allowed them to focus on their observation and express their curiosity.

Another advantage of the remote center that may be relevant is that surgery may have been easier to watch because images were transmitted directly from the operation site. This was hinted at in a report on cadaveric plastic surgery training (7) and could be particularly relevant for MIS involving both thoracoscopy and laparoscopy. This is due to the fact that the live observers, live participants, and the teaching surgeons essentially share the same monitor in the operating room, while the remote observers can observe using several monitors, if available, and engage in open discussions regarding their observations. In fact, remote learning could potentially offer advantages over live observation in an operating room, particularly in situations when several trainees are clamoring for an opportunity to observe.

This simple study identified the potential value of remote education by providing data reflecting the appreciation and satisfaction experienced after a remote CST session. The findings suggest that remote education may be a valid modality for learning, although it is difficult to make specific conclusions regarding the potential value of remote education for learning physical skills due to the subjective nature of this study. Further research is required to determine how effective CST and remote CST are for preparing more experienced surgical trainees with previous experience and exposure to operating rooms and surgical procedures. Of particular interest would be assessing whether CST or remote CST influenced the progress of surgical training.

Based on the data obtained in the present study, while the results for the remote observers and live observers/live participants were similar, there were notable differences and discrepancies in certain criteria, indicating the existence of potential areas for improvement in order to enhance the effectiveness of CST as a learning experience. By identifying areas requiring more effort, the focus of planning for future medical education can be adjusted to overcome shortcomings and potentially include supplementary topics such as experimental surgery or instruction in surgical techniques using models.

As a baseline study for assessing the factors related to a successful remote learning, the favorable reaction of the remote observers would suggest that further research is worthwhile. In addition, the current study could be considered as a trial of the potential of remote learning. With the utilization of Juntendo's existing facilities and the development of a dedicated transmission system tailored for remote teaching, the same training can be conducted with enhanced clarity and broader technical input using more advanced facilities and has the potential for application anywhere, even internationally, with well-renowned surgeons hosting teaching sessions in real time. The legitimate concerns regarding security of information transfer and privacy require the involvement of expert technicians. However, the potential for expanding surgical training from the traditional “see one, do one, teach one” approach to a global interface using remote learning presents an exciting opportunity for every surgeon, no matter how well-renowned. While the scope of the current study is small, the education strategy that could develop based on the data presented could contribute to reducing gaps in surgical education through collaboration among different centers or institutes without any restrictions based on distance.

## Data Availability

The raw data supporting the conclusions of this article will be made available by the authors, without undue reservation.

## References

[1] Zimmerman H Latifi R Dehdashti B Ong E Jie T Galvani C Intensive laparoscopic training course for surgical residents: program description, initial results, and requirements. Surg Endosc. (2011) 25(11):3636–41. 10.1007/s00464-011-1770-6 21643881

[2] La Torre M Caruso C. The animal model in advanced laparoscopy resident training. Surg Laparosc Endosc Percutan Tech. (2013) 23(3):271–5. 10.1097/SLE.0b013e31828b895b 23751991

[3] Luks FI Peers KH Deprest JA Lerut TE. Gasless laparoscopy in infants: the rabbit model. J Pediatr Surg. (1995) 30(8):1206–8. 10.1016/0022-3468(95)90023-3 7472984

[4] Ahmed H Mohammed A Elghazaly H. COVID-19 and medical education. Lancet Infect Dis. (2020) 20(7):777–8. 10.1016/S1473-3099(20)30226-7 32213335 PMC7270510

[5] Artibani W Ficarra V Challacombe BJ Abbou CC Bedke J Boscolo-Berto R EAU policy on live surgery events. Eur Urol. (2014) 66(1):87–97. 10.1016/j.eururo.2014.01.028 24560818

[6] Duty B Okhunov Z Friedlander J Okeke Z Smith A. Live surgical demonstrations: an old, but increasingly controversial practice. Urology. (2012) 79(5):1185.e7–11. 10.1016/j.urology.2011.12.037 22365455

[7] Lisiecki JL Johnson SP Grant D Chung KC. Learners responses to a virtual cadaver dissection nerve course in the COVID era: a survey study. Arch Plast Surg. (2022) 49(5):676–82. 10.1055/s-0042-1756351 36159381 PMC9507583

[8] Yoshida S Miyano G Tanaka M Ikegami M Kato H Seo S Cadaver training for minimally invasive pediatric surgery: a preliminary report. J Laparoendosc Adv Surg Tech A. (2021) 31(12):1485–90. 10.1089/lap.2021.0333 34846942

[9] Thiel W. Supplement to the conservation of an entire cadaver according to W. Thiel. Ann Anat. (2002) 184(3):267–9. 10.1016/S0940-9602(02)80121-2 12061344

[10] Brunckhorst O Challacombe B Abboudi H Khan MS Dasgupta P Ahmed K. Systematic review of live surgical demonstrations and their effectiveness on training. Br J Surg. (2014) 101(13):1637–43. 10.1002/bjs.9635 25312488

